# Analysis of serum immune markers in seropositive and seronegative rheumatoid arthritis and in high-risk seropositive arthralgia patients

**DOI:** 10.1038/srep26021

**Published:** 2016-05-18

**Authors:** Paulina Chalan, Johan Bijzet, Anke van den Berg, Joost Kluiver, Bart-Jan Kroesen, Annemieke M. H. Boots, Elisabeth Brouwer

**Affiliations:** 1 Department of Rheumatology and Clinical Immunology, University of Groningen, University Medical Center Groningen, Groningen, the Netherlands; 2 Groningen Research initiative on healthy Ageing and Immune Longevity (GRAIL) University of Groningen, University Medical Center Groningen, Groningen, the Netherlands; 3 Department of Pathology and Medical Biology, University of Groningen, University Medical Center Groningen, Groningen, The Netherlands; 4 Department of Laboratory Medicine, University of Groningen, University Medical Center Groningen, Groningen, the Netherlands

## Abstract

Presence of autoantibodies precedes development of seropositive rheumatoid arthritis (SP RA) and seropositive arthralgia patients (SAP) are at risk of developing RA. The aims of the study are to identify additional serum immune markers discriminating between SP and seronegative (SN) RA, and markers identifying high-risk SAP. Sera from SAP (n = 27), SP RA (n = 22), SN RA (n = 11) and healthy controls (n = 20) were analyzed using the Human Cytokine 25-Plex Panel. Selected markers were validated in independent cohorts of SP RA (n = 35) and SN RA (n = 12) patients. Eleven of 27 SAP developed RA within 8 months (median follow-up time, range 1–32 months), and their baseline serum markers were compared to 16 non-progressing SAP. SAP and SP RA patients showed a marked overlap in their systemic immune profiles, while SN RA showed a distinct immune profile. Three of 4 markers discriminating between SP and SN RA (IL-1β, IL-15 and Eotaxin, but not CCL5) were similarly modulated in independent cohorts. SAP progressing to RA showed trends for increases in IL-5, MIP-1β, IL-1RA and IL-12 compared to non-progressing SAP. ROC analysis showed that serum IL-5 most accurately discriminated between the two SAP groups (AUC > 0.8), suggesting that baseline IL-5 levels may aid the identification of high-risk SAP.

Rheumatoid arthritis (RA) is a chronic autoimmune disease characterized by inflammation of the synovial membrane. Synovial hyperplasia, neoangiogenesis and invasion of activated innate and acquired immune cells leads to an irreversible destruction of the bone and cartilage of the joint. Aggressive treatment very early in the course of the disease has proven effective in prevention of radiographic progression and tissue damage^1^,^2^,^3^. Based on these observations, postponing or even preventing RA development might become feasible by intervening before the onset of all clinical symptoms of RA^4^. First-degree relatives of RA patients and seropositive arthralgia patients (SAP) have been suggested to represent groups at high risk of RA development and may thus be eligible for preventive intervention^5^.

Seropositivity for autoantibodies such as anti-cyclic citrullinated peptide antibodies (ACPA) and/or rheumatoid factor (RF) is part of the diagnostic criteria for RA^6^. Moreover, ACPA and RF levels have a positive predictive value for future RA development and were detected in serum samples up to 18 years before RA diagnosis^7^,^8^,^9^,^10^,^11^. These autoantibodies may have a direct pathogenic effect in RA. *In vitro*, ACPA-containing immune complexes induced production of pro-inflammatory cytokines via FcγR-dependent triggering of macrophages^12^,^13^ and presence of IgM RF augmented this process^14^. In the pre-clinical stage of RA, emergence of ACPA and RF or increase of ACPA reactivity preceded the elevation of serum cytokine levels^9^. It has been suggested that different inflammatory pathways are involved in the development of seronegative (SN RA) and seropositive RA (SP RA). Presence of autoantibodies in early RA has been shown to confer risk of more aggressive, progressive and erosive disease^15^,^16^,^17^,^18^,^19^,^20^. SP RA patients have a greater need for disease-modifying anti-rheumatic drugs (DMARDs) or aggressive treatment^16^,^17^ and a lower chance of achieving drug-free remission^20^,^21^. Furthermore, presence of ACPA or RF has been associated with the development of comorbidities, such as vasculitis^22^ and pulmonary diseases^23^. Worse clinical outcome suggested increased inflammatory responses in seropositive RA and prompted analysis of the local inflammatory site^24^,^25^,^26^,^27^. Data on the differences in the systemic inflammatory markers between SP and SN RA is limited^28^.

In the present study, we aimed to identify serum immune markers that could discriminate between recently diagnosed SP RA and SN RA patients. Selected markers were evaluated in independent cohorts of SP and SN RA patients. Secondly, we aimed to identify baseline serum markers in SAP that could discriminate between SAP who progressed to RA and SAP who did not progress to RA.

## Results

### Description of study cohorts

The SAP group was characterized by a significantly lower CRP (p = 0.0055 and p = 0.0005, respectively), ESR (p = 0.010 and p < 0.0001, respectively) and TJC (p = 0.0002 and p = 0.0071, respectively) when compared to SP RA and SN RA patients (Table 1). The percentages of ACPA and RF single- and double-positive patients were similar in the SAP and SP RA patient groups, with the majority being double-positive (ACPA + RF+). Comparison of SP with SN RA patients showed no differences between the baseline characteristics such as age, sex, duration of symptoms until RA diagnosis, CRP, ESR, DAS28, SJC, TJC or the frequency of patients with radiographic changes (Table 1). The independent cohorts of recently diagnosed DMARD-naïve seropositive (n = 35) and seronegative (n = 12) RA patients included in the validation study did not differ in age, sex, symptom duration until RA diagnosis, CRP, ESR, TJC, SJC, DAS28 and presence of erosions (Supplementary Table S1). Demographical and clinical characteristics of the SP and SN RA patients in the independent cohorts were similar, although age and ESR were lower (p = 0.039 and p = 0.029, respectively) in the second SN RA cohort (Supplementary Table S1). Comparison of the baseline demographical/clinical characteristics of SAP progressing to RA (SAP = > RA) and SAP showed no differences between the groups. SAP who developed RA tended to be older at the inclusion of the study, compared to SAP not progressing during the follow-up (p = 0.058; Supplementary Table S2).

### Unsupervised hierarchical analysis of serum immune markers separates SAP and SP RA from SN RA and HC

**Figure 1:**
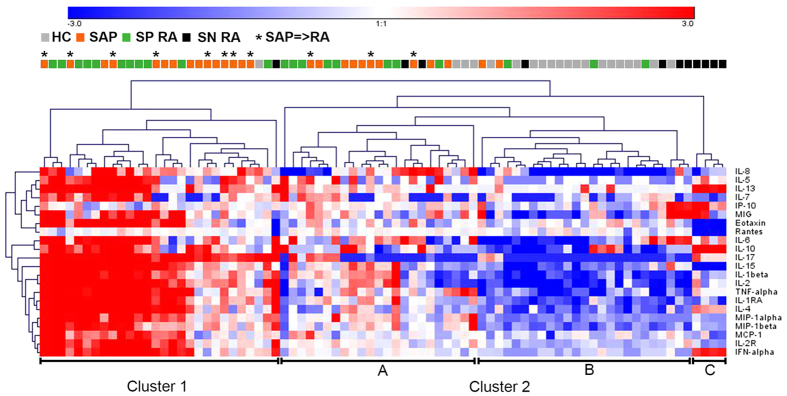
Unsupervised hierarchical clustering analysis of serum markers from HC, SAP, SP RA and SN RA. Unsupervised hierarchical clustering (average linkage method, Euclidean distance metric) of the log2-transformed data of 22 serum markers measured in 20 HC, 27 SAP, 22 SP RA and 11 SN RA patients. Asterisks indicate SAP who progressed to RA (SAP = > RA) during follow-up.

ANOVA of the 4 study groups: HC, total SAP, SP RA and SN RA revealed significant differences (p ≤ 0.002) for 22 out of the 25 markers analyzed. IL-12, IFN-γ and GM-CSF were not significantly different between the study groups. Unsupervised hierarchical clustering of the 22 significant markers revealed a separation into 2 clusters (Fig. 1). Fifty-six percent of all SAP and 50% of the SP RA patients form the vast majority of individuals in cluster 1 that is characterized by a higher expression of the 22 markers analyzed. Cluster 2 consisted of three subgroups with 37% of the remaining SAP and 36% of the SP-RA patients grouping together in cluster 2A (intermediate expression levels) and most HC (80%) clustering together in cluster 2B (relative low expression). Cluster 2C was characterized by intermediate expression of the serum immune markers and included 36% of the SN RA patients. The remaining SN RA patients were dispersed among all other clusters. Interestingly, 8/11 SAP who later developed RA (SAP = > RA) were included in cluster 1 (indicated by asterisks).

**Figure 2:**
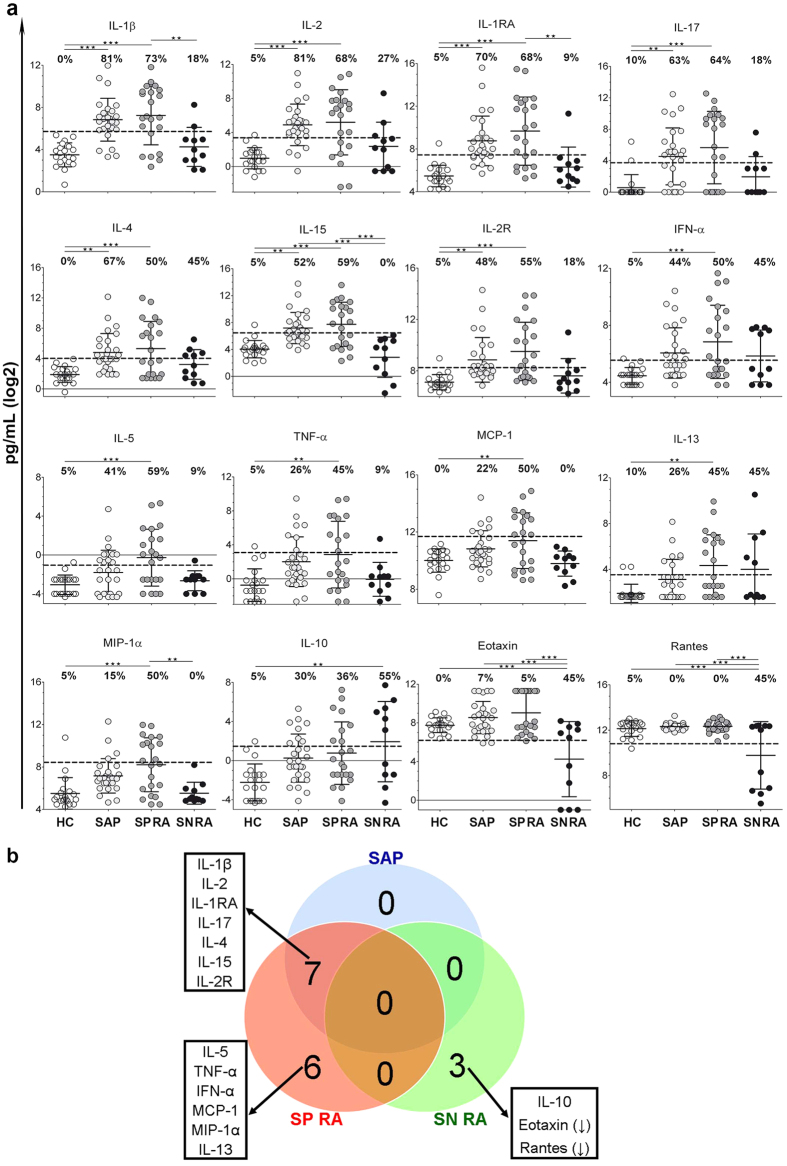
Serum markers in patient groups compared to HC. (**a**) Graphs depict expression levels of log2-transformed values in HC, SAP, SP RA and SN RA. The dotted line indicates the threshold of mean ± 2 SD of HC values. Horizontal lines represent mean and whiskers represent SD. Percentages above the data sets indicate the frequency of subjects showing expression values above/below the threshold. Differences between the groups were calculated using ANOVA and post-hoc Tukey’s test with p ≤ 0.002 regarded as statistically significant after the Bonferroni correction. Significance indicated as *** for p ≤ 0.0005 and ** for p ≤ 0.002. (**b**) Venn diagram showing differences and overlap in serum markers that were 1) statistically different between patient groups when compared to HC, and 2) increased/decreased above/below mean ± 2 SD of HC values in ≥45% SAP, SP RA or SN RA.

In order to identify the most pronounced markers per group, we selected markers that showed an increase/decrease in expression of more than the mean ± 2SD of the HC levels in at least 45% of patients. Sixteen out of 22 markers, showed elevated or decreased levels in ≥45% of patients of at least one group (Fig. 2a). The overlap and differences of the significantly increased or decreased markers in ≥45% of SAP/SP RA/SN RA are visualized in a Venn diagram (Fig. 2b). All markers with increased levels in SAP were also increased in SP RA, i.e. IL-1β (81% and 73%, respectively), IL-2 (81% and 68%), IL-1RA (70% and 68%), IL-17 (63% and 64%), IL-4 (67% and 50%), IL-15 (52% and 59%), and IL-2R (48% and 55%). The markers that showed a pronounced upregulation in SP RA but not in the other groups were IL-5 (59%), MCP-1 (50%), MIP-1α (50%), IFN-α (50%), TNF-α (45%) and IL-13 (45%). IL-10 serum levels were increased above the cut-off only in SN RA patients (55%). Next to the pronounced increase of IL-10, SN RA patients had decreased levels of Eotaxin and Rantes in 45% of patients. These decreases were not observed in the other groups (Fig. 2).

### Validation of serum immune markers in independent SP RA and SN RA cohorts

To verify the differences between early SP RA and SN RA patients, we repeated the measurement of a selected (see “Statistical analysis” for the selection criteria) set of serum markers (IL-1β, IL-15, Eotaxin, Rantes) in independent SP RA and SN RA cohorts (Table 2). Significantly higher levels of IL-1β (p = 0.0125), and trends for an increase of IL-15 and Eotaxin (p = 0.0339 and p = 0.0233, respectively, Table 2) were observed in SP RA compared to SN RA. The decreased levels of Rantes in SN RA compared to SP RA could not be confirmed.

### Baseline levels of serum markers identifying high-risk SAP

**Figure 3:**
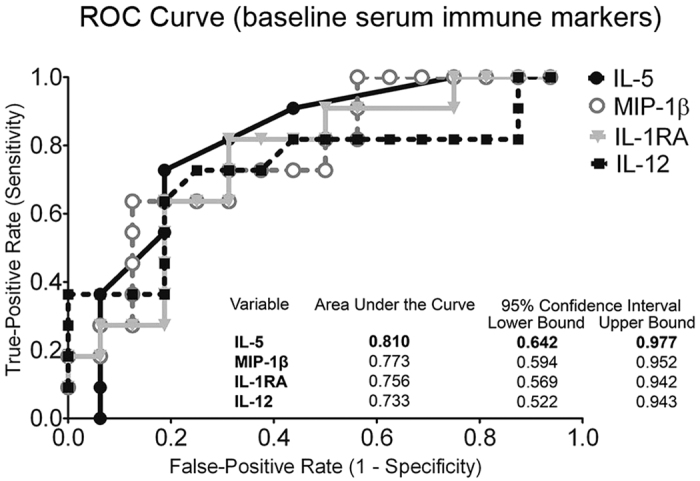
Receiver operating characteristic curves (ROC) for selected baseline serum markers in SAP = > RA and SAP not progressing. ROC analysis and area under the curve of ROC curves was performed for 4 immune markers whose baseline levels showed trends towards a significant difference in the comparison of SAP = > RA and SAP not progressing (as demonstrated in Table 3).

We investigated whether the baseline serum markers differed between SAP who progressed to RA (SAP = > RA, median time to arthritis development was 8 [range 1–32] months) and SAP who did not progress to RA during the follow-up period (median follow-up time was 26 [range 6–33] months). Eleven of the 27 (41%) SAP progressed to SP RA (Fig. 1, Supplementary Table S2). SAP = > RA were characterized by higher baseline levels of IL-5, MIP-1β, IL-1RA and IL-12, compared to SAP who did not progress to RA (Table 3). However, when corrected for multiple comparisons (p ≤ 0.002) only trends for the increases in baseline IL-5, MIP-1β, IL-RA and IL-12 in SAP = > RA were noted (p = 0.007, p = 0.019, p = 0.028, p = 0.046, respectively). Receiver operating characteristic (ROC) analysis was used to determine if baseline levels of any of these 4 immune markers may discriminate between SAP who progress to RA from SAP who do not. A good discriminatory ability (Area Under the Curve, [AUC] > 0.8) was obtained for IL-5 (Fig. 3). Our data suggest that baseline IL-5 levels may help to identify SAP at risk for future RA development.

## Discussion

The aims of the present study were to compare serum immune markers for their ability to discriminate between early SP and SN RA; and to identify serum immune markers that may predict progression to RA in SAP.

It has been suggested that RA does not begin at the level of the joint but is preceded by systemic inflammation^9^. This is supported by several retrospective studies that demonstrated systemic elevation of various inflammatory factors in the pre-RA stage^10^,^11^,^29^. Analysis of the markers of systemic inflammation in SAP, who are at risk of RA development^5^, has not yet been performed in a prospective study. Analysis of the local inflammation in SAP showed either weak^30^,^31^ or lack of^32^ signs of subclinical synovitis in SAP.

One of the conclusions of the present study is that the increase in markers of systemic inflammation is also a feature of SAP, and that the SAP immune profile is highly similar to the profile seen in SP RA patients. The marked overlap of serum markers in SAP and SP RA reflects a common inflammatory background between both conditions with increased levels of IL-1β, IL-1RA, associated with general inflammation; increased levels of T-cell activation markers (IL-2, IL-2R, IL-4) and increased levels of markers associated with Th17-specific activation (IL-17, IL-1β, IL-15). IL-1β levels were elevated in most SAP and SP RA patients. This was mirrored by elevations in IL-1RA. The concomitant increase of IL-1β and IL-1RA indicates activation of both pro- and anti-inflammatory pathways. Despite the observed increase of IL-2, known to promote Th1 and Treg cells and inhibit Th17 differentiation^33^,^34^, no alterations of Th1-type cytokines (IFN-γ, IL-12) or the Treg-associated IL-10 were observed in SAP and SP RA. In contrast, IL-17 was significantly increased in these two SP groups. Thus, our results undermine the notion of RA as a Th1-mediated disease and support a role of Th17 cells in the early stages of SP RA pathogenesis, as previously suggested by others^24^,^25^. Moreover, increased levels of IL-1β and IL-15 in the periphery of SAP and SP RA may contribute to maintaining pathogenic Th17 responses, as they have been demonstrated to promote Th17 differentiation^35^ and trigger IL-17 expression^36^, respectively.

The second conclusion from the present study is that, in contrast to SAP and SP RA, SN RA patients showed a distinct immune marker profile. We have identified (main study) and confirmed (validation study) IL-1β as an immune marker differentially expressed in SP RA and SN RA. This observation suggests that the pathological pathways involving blood monocytes may be activated in seropositive but not seronegative RA, as IL-1β has been reported to be expressed by this cell type (as well as tissue macrophages and dendritic cells) in response to stimulation^37^. Also, IL-15 and Eotaxin may be useful in discriminating between SP and SN RA as these markers, but not CCL5, were similarly modulated in the independent cohorts.

Despite the differences in pro- and anti-inflammatory markers between SP RA and SN RA, clinical features of these groups at baseline were similar. Most available studies showed that, in line with our cohorts, all or most of the baseline demographical and clinical characteristics were similar between ACPA+ and ACPA- RA patients^16^,^17^,^24^,^26^,^27^. However, significantly increased CRP, ESR and DAS28 levels, and increased radiographic damage in ACPA+ patients have also been reported^16^,^18^,^19^,^26^. It has been suggested that differences in the pathogenesis and prognosis between SP RA and SN RA are the consequence of different pathological events at the inflammatory site. However, most studies reported similar levels of inflammatory markers in the joints of SP RA and SN RA, with significantly increased levels being observed only for CCL20, IL-10, IL-1β and IL-17^24^,^25^ in ACPA+ RA. Increased lymphocytic infiltration, expression of T-cell markers and lymphocyte chemoattractant in the synovium of ACPA+ compared to ACPA− RA patients has been reported^26^. These differences in synovial infiltration between ACPA+ and ACPA− RA patients, however, were not confirmed by three other studies^24^,^25^,^27^. Also, the numbers of B-cells, plasma cells in the synovium^24^,^26^,^27^ or B-cells in synovial fluid and blood^38^ were found to be similar between seropositive and seronegative RA. Thus, there is no consensus on synovial markers discriminating between SP and SN RA. Our study is the first to describe specific differences in serum immune markers in SP RA and SN RA. Deane *et al.* reported that the percentage of pre-diagnosis samples positive for cytokines was lower in patients who later developed SN RA as compared to the percentage of cytokine positive samples in patients who later developed SP RA^28^. ACPA/RF-containing immune complexes can trigger cytokine production via FcγR-crosslinking, as demonstrated *in vitro*^12^,^13^,^14^. We hypothesize that this mechanism is responsible for the observed more pronounced expression of serum markers in SP RA compared to SN RA. The qualitative differences between SP RA and SN RA indicate the importance of stratifying RA patients according to the autoantibody status in studies investigating pathological pathways involved in RA and in clinical trials.

It is well known that rheumatoid factor, particularly IgM-RF, may interfere with the assay outcome by false-positive binding. Therefore, we explored this issue by measuring levels of several immune markers in a serum sample with high RF level, before and after RF precipitation using polyethylene glycol (PGE 6000)^39^. RF blocking had limited effects on the detection of the cytokines tested (data not shown). Thus, similar to others^29^, we decided to not incorporate the RF blocking step in our procedures. However, possible interference with RF can thus not be fully excluded and is a limitation of the current study.

The third conclusion of this study is that baseline levels of IL-5 may aid in identifying high risk SAP. The percentage of SAP who developed RA in our cohort was similar to that reported by others^30^,^40^,^41^. The role of IL-5 in RA; a Th2-specific cytokine primarily involved in regulation of eosinophil functions in the tissue^42^, is ill-defined. IL-5 was not present in the synovium and rheumatoid nodules of RA patients^43^,^44^. Implications of the increase of systemic IL-5 levels in SAP = > RA, a serum marker that was also found elevated in 59% of SP RA, would require further studies. So far, the identification of high risk SAP relied mostly on demographic (i.e. presence of the first-degree relative with RA, alcohol non- use) and clinical variables (i.e. duration of the morning stiffness ≥1 hour, symptoms and VAS pain ≥50)^40^. Recently, the combination of a type I IFN signature with a B cell^low^ signature was found to predict RA development in SAP^40^,^45^. Our data suggest that measurement of serum IL-5 may add to current prediction models.

## Methods

### Subjects

In this comparative study we included 22 recently diagnosed SP RA (ACPA+ and/or RF+) patients; 11 recently diagnosed SN RA (ACPA− and RF−) patients; 27 SAP and 20 healthy controls (HC, Table 1). For validation purposes, we included serum samples from 2 independent cohorts of SP RA (n = 35) and SN RA (n = 12, Supplementary Table S1). Inclusion criteria for the prospective SAP cohort, other than seropositivity, were the presence of arthralgia (tender joint count [TJC] ≥ 1) but no diagnosis of arthritis (swollen joint count [SJC] = 0). The diagnosis of seropositive arthralgia was made by a trained rheumatologist (EB), after patients with joint complaints were referred to our early arthritis clinic or early arthritis recognition clinic by their general practitioner. SAP were seen every 6 months or had a visit scheduled when their joint complaints progressed, including swelling of the joints. Upon diagnosis of arthritis the prospective follow-up in the SAP cohort was terminated. Early RA patients, fulfilling the 1987 or 2010 American College of Rheumatology (ACR) classification criteria for RA were included at time of diagnosis and these patients did not receive disease modifying anti-rheumatic drugs (DMARDs). Both SAP and RA were treated with non-steroidal anti-inflammatory drugs (NSAIDs) only. At the time of inclusion recently diagnosed RA and SAP were assessed for the presence of radiographic damage. Healthy subjects were not recently vaccinated, did not have an infection and did not use immunosuppressive drugs at the time of blood withdrawal, as assessed by a health questionnaire. All participants gave their informed consent and the study was approved by the local medical ethics committee (UMC Groningen). All experimental protocols were carried out in accordance with the approved guidelines and were approved by the ethical committee of UMC Groningen.

Demographical and clinical characteristics of all study participants are shown in Tables 1, Supplementary Tables S1 and S2. Eleven of the SAP (41%) progressed to RA (indicated as SAP = > RA) over a median follow-up of 8 (range 1–32) months. The median follow-up time for the non-progressing SAP (until the last visit or until December 2014) was 26 (range 6–33) months.

ACPA serum levels were determined by anti-IgG CCP fluorescent enzyme immunoassay using Phadia 250 System (Thermo Fisher Scientific, Uppsala, Sweden) and serum levels ≥ 10 IU/ml were considered as positive. Total RF serum levels were determined by turbidimetry using a modular analyzer (Roche, Mannheim, Germany) and serum levels ≥ 15 IU/ml were considered positive.

### Measurement of serum immune markers

Peripheral blood was collected in anticoagulant-free tubes, centrifuged (at 1200 g for 10 min) and serum was stored at −20 °C until analysis. Serum immune markers were quantified with the Human Cytokine 25-Plex Panel (Life Technologies, Carlsbad, CA, USA) according to the manufacturer’s instructions. Custom-made Luminex immunoassay (Life Technologies) was used for the detection of IL-1β, IL-15, Eotaxin and Rantes in the validation cohorts. Samples were measured using Luminex 100 System (Luminex, Austin, Tx, USA) and data were analyzed with StarStation software, version 2.3 (AppliedCytometry, Birmingham, UK). The following markers were assessed in the main study: IL-1β, IL-2, IL-4, IL-5, IL-6, IL-7, IL-10, IL-12 (p40/p70), IL-13, IL-15, IL-17, IFN-α, IFN-γ, GM-CSF, TNF-α, IL-1 receptor antagonist (IL-1RA), IL-2 R, Eotaxin (CCL11), IL-8, IP-10 (CXCL10), MCP-1 (CCL2), MIG (CXCL9), MIP-1α (CCL3), MIP-1β (CCL4) and Rantes (CCL5).

### Statistical analysis

Demographical and clinical characteristics were compared with ANOVA or Kruskall-Wallis test for continuous data with normal and non-normal distribution, respectively. Categorical data were analyzed using chi-squared test. Data obtained from 2 groups were compared with Mann-Whitney or Fisher’s exact tests. P < 0.05 was considered statistically significant. For all analyses, data were log2-transformed in order to approach a Gaussian distribution. Differences between the groups were analyzed with ANOVA and a Tukey’s post-hoc test. Differences between the 2 SAP groups were compared using Mann-Whitney test. In order to adjust for multiple comparisons, results were considered statistically significant when p ≤ 0.002 (Bonferroni correction). Cytokines for the validation study were chosen according to the following criteria: 1) their levels were significantly different between SP RA and SN RA in the main cohort, 2) ≥45% of SP RA and SN RA patients showed expression levels above or below mean ±2 standard deviations (SD) of the HC values and 3) size of the independent sample cohort required to obtain the desired power (1-sided, sensitivity 90%, confidence intervals 95%) was sufficient. Differences between the groups of SP RA and SN RA from the independent cohorts or from the main cohorts were compared using Mann-Whitney test (after multiple testing correction, p ≤ 0.0125 was considered statistically significant). Analyses were performed with IBM SPSS Statistics 20 (SPSS, Chicago, IL, USA). Hierarchical clustering analysis was done with Genesis 1.7.6 software^46^ using Euclidean distances and average linkage.

## Additional Information

**How to cite this article**: Chalan, P. *et al.* Analysis of serum immune markers in seropositive and seronegative rheumatoid arthritis and in high-risk seropositive arthralgia patients. *Sci. Rep.* **6**, 26021; doi: 10.1038/srep26021 (2016).

## Supplementary Material

Supplementary Information

## Figures and Tables

**Table 1:** Baseline demographical and clinical characteristics of the subjects included in the study.

	HC	SAP	SP RA	SN RA
N	20	27	22	11
Age [yrs]; mean (SD)	55.7 (7.5)	50.8 (14.4)	53.4 (12.3)	60.3 (7.5)
Gender; % female (n)	65.0 (13)	66.7 (18)	68.2 (15)	72.7 (8)
Symptom duration [mos]; median (range)	–	24 (1–33)	6 (1–84)	5 (1–13)
ACPA positive; % (n)	NR	92.6 (25)	90.9 (20)	0.0 (0)
RF positive; % (n)	15.0 (3)	88.9 (24)	81.8 (18)	0.0 (0)
CRP [mg/l]; median (range)	NR	5.0 (5.0–29.0)*	12.5 (5.0–75.0)	17.0 (5.0–57.0)
ESR [mm/h]; median (range)	NR	12.0 (2.0–43.0)*	21.0 (2.0–96.0)	45.0 (22.0–88.0)
TJC [n]; median (range)	NR	1.0 (0.0–16.0)*	7.0 (0.0–23.0)	5.0 (0.0–27.0)
SJC [n]; median (range)	NR	0.0 (0.0–0.0)	6.0 (0.0–16.0)	4.0 (0.0–14.0)
DAS28; mean (SD)	NR	NR	4.9 (1.6)	5.0 (1.4)
Erosions; % (n)	NR	-	13.6 (3)	18.2 (2)

HC: Healthy controls; SAP: Seropositive arthralgia patients; SP RA: Seropositive rheumatoid arthritis patients; SN RA: Seronegative rheumatoid arthritis patients; mos: months; ACPA: Anti-cyclic citrullinated proteins antibodies; RF: Rheumatoid factor; CRP: C-Reactive protein; ESR: Erythrocyte sedimentation rate; TJC: Tender joint count; SJC: Swollen joint count; DAS28: Disease activity score 28; NR: Not reported: *Indicates p < 0.05.

**Table 2:** Results of the validation study in independent SP RA and SN RA cohorts.

	Validation cohorts			Main cohorts		
	SP RA	SN RA	p-value	SP RA	SN RA	p-value
N	35	12	–	22	11	–
IL-1β [pg/mL(log2)]; median (IQR)	4.83 (1.01)	4.35 (0.37)	**0.0125**	7.10 (4.49)	3.98 (1.96)	**0.0044**
IL-15 [pg/mL(log2)]; median (IQR)	4.82 (0.66)	4.45 (0.55)	**0.0339**	7.95 (6.13)	4.18 (5.03)	**0.0016**
Eotaxin [pg/mL(log2)]; median (IQR)	6.51 (0.70)	5.15 (3.99)	**0.0233**	8.03 (4.20)	6.48 (8.57)	**0.0031**
Rantes [pg/mL(log2)]; median (IQR)	12.99 (0.88)	13.06 (0.38)	0.8836	12.45 (0.63)	12.02 (5.72)	**0.0111**

SP RA: Seropositive rheumatoid arthritis patients; SN RA: Seronegative rheumatoid arthritis patients; IQR: Interquartile range. SP RA and SN RA cohorts from the validation or the main study were compared with Mann-Whitney test. P ≤ 0.0125 was considered statistically significant.

**Table 3:** Baseline levels of serum markers in SAP who progressed to RA and SAP not progressing.

Immune marker	SAP not progressing(n = 16)	SAP = > RA(n = 11)	p-value
	[pg/mL(log2)]; median (IQR)		
**IL-5**	−4.00 (2.66)	−0.54 (1.65)	**0.007**
**MIP-1β**	7.50 (1.77)	8.24 (1.99)	**0.019**
**IL-1RA**	7.61 (1.84)	9.02 (3.61)	**0.028**
**IL-12**	7.95 (0.36)	8.15 (1.41)	**0.046**
IL-2	4.34 (2.81)	5.22 (4.09)	0.109
IL-6	2.23 (3.69)	3.03 (2.13)	0.109
IL-1β	6.66 (2.82)	7.04 (4.25)	0.132
IL-7	3.32 (3.08)	5.06 (4.92)	0.167
IL-2 R	7.96 (1.14)	8.37 (1.87)	0.175
Eotaxin	7.81 (2.10)	8.86 (2.11)	0.199
IL-13	1.68 (1.74)	3.32 (2.18)	0.204
MIP-1α	6.84 (1.33)	6.99 (2.82)	0.267
IFN-α	5.20 (2.54)	5.63 (2.85)	0.275
IL-17	3.70 (7.02)	4.78 (1.98)	0.286
TNF-α	0.98 (4.78)	1.32 (2.83)	0.311
IL-15	6.49 (2.14)	7.03 (4.65)	0.336
IFN-γ	−1.74 (0.74)	−1.00 1.22)	0.388
IL-10	−0.33 (5.47)	0.70 (2.61)	0.401
GM-CSF	4.60 (3.70)	5.00 (1.54)	0.412
MCP-1	10.42 (0.89)	10.69 (2.23)	0.430
IP-10	3.98 (0.73)	4.34 (1.72)	0.570
Rantes	12.32 (0.37)	12.33 (.029)	0.604
IL-8	8.62 (1.87)	9.43 (3.04)	0.639
MIG	3.77 (1.95)	3.12 (2.12)	0.902
IL-4	4.21 (2.40)	3.94 (3.97)	1.000

SAP: seropositive arthralgia patients; IQR: Interquartile range. SAP groups were compared using Mann-Whitney test. P ≤ 0.002 was considered statistically significant.
