# Supplementary material for: Analysis of serum immune markers in seropositive and seronegative rheumatoid arthritis and in high-risk seropositive arthralgia patients
Source: Sci Rep. 2016 May 18;6:26021. doi: 10.1038/srep26021 (PMC4870704; doi:10.1038/srep26021)
Supplement: Supplementary Information [file srep26021-s1.pdf]

Analysis of serum immune markers in seropositive and seronegative rheumatoid arthritis and in high-risk seropositive arthralgia patients

Paulina Chalan, Johan Bijzet, Anke van den Berg, Joost Kluiver, Bart-Jan Kroesen, Annemieke M.H. Boots, Elisabeth Brouwer.

Supplementary Table S1. Baseline demographic and clinical characteristics of the subjects included in the validation study.

	SP RA validation cohort	P-value compared to SP RA main cohort	SN RA validation cohort	P-value compared to SN RA main cohort
N	35	–	12	–
Age [yrs]; mean (SD)	53.9 (10.1)	ns	49.8 (14.2)	0.039
Gender; % female (n)	65.7 (23)	ns	66.7 (8)	ns
Symptom duration [mos]; median (range)	6 (1-6)	ns	5 (1-36)	ns
ACPA positive; % (n)	88.6 (31)	ns	0.0 (0)	–
RF positive; % (n)	100.0 (35)	0.046	0.0 (0)	–
CRP [mg/l]; median (range)	9.0 (5.0-104.0)	ns	5.0 (5.0-63.0)	ns
ESR [mm/h]; median (range)	28.0 (3.0-83.0)	ns	24.5 (3.0-48.0)	0.029
TJC [n]; median (range)	5.0 (0.0-28.0)	ns	8.5 (0.0-24.0)	ns
SJC [n]; median (range)	5.0 (0.0-28.0)	ns	9.0 (0.0-25.0)	ns
DAS28; mean (SD)	4.6 (1.6)	ns	5.8 (2.3)	ns
Erosions; % (n)	27.8 (10)	ns	16.7 (2)	ns

SP RA: seropositive rheumatoid arthritis; SN RA: seronegative rheumatoid arthritis; mos; months; ACPA: anti-cyclic citrullinated proteins antibodies; RF: rheumatoid factor; CRP: C-reactive protein; ESR: erythrocyte sedimentation rate; TJC: tender joint count; SJC: swollen joint

count; DAS28: disease activity score 28; ns = not statistically significant. Groups were compared using Mann-Whitney test. P<0.05 was considered statistically significant.

Supplementary Table S2. Baseline demographic and clinical characteristics of SAP not progressing and SAP=>RA.

	SAP not progressing	SAP=>RA	p-value
N	16	11	
Age [yrs]; mean (SD)	47.3 (12.1)	55.8 (16.4)	0.058
Gender; % female (n)	75.0 (12)	54.5 (6)	0.411
Symptom duration [mos]; median (range)	10 (1-72)	9 (2-60)	0.961
Follow-up time to arthritis development [mos]; median (range)	NA	8 (1-32)	NA
Total follow-up time [mos]; median (range)	26 (6-33)	NA	0.786
ACPA positive; % (n)	87.5 (14)	90.9 (10)	1.0
RF positive; % (n)	87.5 (14)	90.9 (10)	1.0
CRP [mg/l]; median (range)	5.0 (5.0-29.0)	5.0 (5.0-19.0)	0.134
ESR [mm/h]; median (range)	14.0 (2.0-32.0)	11.0 (5.0-43.0)	0.980
TJC [n]; median (range)	1.0 (0.0-11.0)	2.0 (0.0-16.0)	0.248

SAP: seropositive arthralgia patients; mos: months; NA = not applicable; ACPA: anti-cyclic citrullinated proteins antibodies; RF: rheumatoid factor; CRP: C-reactive protein; ESR:

erythrocyte sedimentation rate; TJC: tender joint count. P<0.05 was considered statistically significant.

Supplementary Table S3. Raw values of the immune marker levels in the cohorts used.

Immune marker	Limit of detection (LOD)	HC n=20	SAP not progressing n=16	SAP=>RA n=11	SP RA n=22	SN RA n=11
	pg/mL	median pg/mL (IQR)	median pg/mL (IQR)	median pg/mL (IQR)	median pg/mL (IQR)	median pg/mL (IQR)
**IL-1β**	**0.688**	11.4 (12.2)	101.0 (118.9)	132.0 (1198.8)	137.1 (705.5)	15.8 (23.1)
**IL-1RA**	**4.585**	38.0 (34.4)	195.4 (276.2)	520.7 (2920.3)	774.5 (5392.2)	67.3 (86.6)
**IL-2R**	**0.215**	128.0 (52.6)	249.0 (272.5)	332.0 (745.1)	386.5 (2723.9)	147.1 (165.1)
**TNF-α**	**0.152**	0.4 (0.7)	2.0 (22.4)	2.5 (8.9)	3.9 (143.7)	1.2 (0.8)
**IL-6**	**0.104**	1.3 (5.5)	4.8 (16.2)	8.2 (18.6)	25.2 (131.6)	20.6 (34.3)
**IL-2**	**0.201**	1.9 (2.1)	20.4 (34.8)	37.2 (258.5)	92.6 (211.0)	4.5 (11.5)
**IL-15**	**0.651**	15.5	90.2 (176.6)	130.8	248.4	18.1

		(11.6)		(1280.9)	(1503.5)	(40.4)
**IFN-α**	**1.591**	23.0 (14.8)	36.7 (122.4)	49.5 (227.4)	50.4 (520.7)	30.7 (200.2)
**MCP-1 (CCL-2)**	**9.303**	1108.0 (824.8)	1369.0 (900.0)	1657.0 (3374.9)	3064.0 (7950.3)	1131.0 (697.3)
**MIP-1α (CCL3)**	**0.502**	30.8 (22.8)	114.3 (100.5)	127.2 (538.7)	349.4 (1324.0)	32.0 (34.0)
**MIP-1β (CCL4)**	**0.584**	52.1 (44.5)	181.2 (179.4)	302.8 (554.9)	249.6 (1014.9)	68.4 (67.5)
**IP-10 (CXCL10)**	**0.091**	11.8 (8.3)	15.8 (9.1)	20.3 (31.3)	16.7 (12.5)	28.7 (95.9)
**MIG (CXCL9)**	**2.851**	2.3 (4.4)	13.7 (19.0)	8.7 (28.3)	13.8 (96.7)	5.1 (74.5)
**IL-8**	**0.262**	32.8 (142.2)	394.7 (713.2)	692.1 (2070.9)	462.3 (1244.2)	160.7 (1112.8)
**Rantes (CCL5)**	**0.866**	5387.0 (3221.0)	5115.0 (1332.0)	5159.0 (1016.0)	5599.0 (2350.0)	4156.0 (5187.9)
**IL-7**	**1.420**	12.4 (29.8)	14.5 (29.8)	33.3 (117.4)	51.8 (165.5)	8.0 (30.0)
**GM-CSF**	**1.998**	6.7 (9.4)	24.6 (56.5)	31.9 (36.4)	14.1 (146.8)	8.6 (29.7)
**IL-12**	**0.832**	120.4 (42.2)	246.1 (59.7)	283.3 (423.9)	337.0 (312.4)	198.7 (4317.0)

**IFN-γ**	**0.594**	0.3 (0.4)	0.3 (0.2)	0.5 (0.4)	0.3 (0.4)	0.3 (0.4)
**IL-4**	**0.421**	3.7 (3.3)	18.8 (37.1)	15.4 (88.0)	24.2 (361.3)	8.4 (23.9)
**IL-5**	**0.015**	0.2 (0.1)	0.1 (0.2)	0.7 (0.8)	1.0 (3.7)	0.2 (0.1)
**IL-13**	**0.440**	3.0 (0.4)	3.2 (7.0)	10.0 (20.4)	7.9 (93.8)	3.4 (104.4)
**Eotaxin (CCL11/24/26)**	**0.062**	223.1 (161.2)	224.4 (456.3)	463.1 (655.8)	262.0 (2372.4)	89.4 (189.3)
**IL-10**	**0.015**	0.2 (0.3)	0.9 (7.9)	1.6 (3.3)	1.0 (9.1)	8.8 (40.6)
**IL-17**	**5.025**	1.0 (0.0)	13.3 (158.9)	27.4 (64.0)	164.6 727.6)	1.0 (7.0)

HC: healthy controls; SAP: seropositive arthralgia patients; SP RA: seropositive rheumatoid arthritis patients; SN RA: seronegative rheumatoid arthritis patients; IQR: Interquartile range.
